# An Extraordinary Voice Expressed Through Humor: A Tribute to Casey Quinlan

**DOI:** 10.2196/54527

**Published:** 2023-12-12

**Authors:** Susan S Woods, Jan Oldenburg, Daniel van Leeuwen, Jane Sarasohn-Kahn, Matthew F Hudson

**Affiliations:** 1 Tufts University School of Medicine Boston, MA United States; 2 Participatory Health Consulting Richmond, VA United States; 3 Health Hats Boston, MA United States; 4 Think-Health LLC Philadelphia, PA United States; 5 Prisma Health Greenville, SC United States

**Keywords:** participatory medicine, co-design, co-production, patient engagement, patient empowerment, electronic health record, patient portal, open notes, evidence-based medicine, shared decision-making

## Abstract

The *Journal of Participatory Medicine* introduces *Extraordinary Lives*, a new journal section celebrating the voices and work of steadfast advocates of participatory medicine that we have lost. This inaugural essay spotlights Casey Quinlan, a patient activist who effectively used her humor and incisive analysis of health care to encourage others to strive for meaningful change. A first-generation “professional patient,” Casey served as a role model who inspired many to share their stories and achieve genuine partnerships in care delivery. A maker of “good trouble,” her voice and stance were part of her power and influence in disrupting the status quo. We present her fight for personal access to health data, her aspiration for personally customized evidence, and her drive for all people to control their health and their health care.

## Introduction

The *Journal of Participatory Medicine* is introducing a new journal section called *Extraordinary Lives.* Papers in this category are essays celebrating and commemorating advocates and pioneers the world has lost, yet who have left a lasting impact on participatory medicine. These essays lift a torch to champions who contributed to transitioning health care from a top-down, revenue-driven enterprise [1] to one exhibiting genuine partnership between patients and professionals delivering care.

Our journal’s mission is to generate knowledge on co-design to improve health care and research, and to demonstrate how the internet and digital services enable people to achieve healthy lives [2]. Our field is dependent upon and indebted to advocates who contribute to these aims. To inaugurate *Extraordinary Lives*, we highlight the life and work of Casey Quinlan (1952-2023). Many have written about her wisdom, insights, and contributions [3,4]. Through her unquenchable activism targeted across many participatory care domains, Casey is an exemplar to launch such a tribute. Here, we describe only a few of her numerous contributions; we focus attention on her efforts in advancing personal access to health data, activating patients to fully participate in their care, and encouraging patients and caregivers to contribute their expertise across the health care ecosystem.

## Performer, Journalist, Blogger, Podcaster, and Activist

Casey was born Mary Martha Casey at the US Naval Academy in Annapolis, Maryland, to Martin Michael Casey and Marie Elizabeth Rodgers Casey. Her father was a Navy captain and her grandfather was a Navy rear admiral; unsurprisingly, Casey considered herself a warrior throughout her life. Early on, she dropped Mary as her first name and substituted Casey, subsequently taking Quinlan as her last name in honor of her paternal grandmother.

Raised on both the west and east coasts of the United States and in England, she gained a broad worldview that helped shape a skeptical perspective on institutions. In grade school, she adopted the moniker “Mighty Casey” after being teased on the playground about “Casey at the bat” striking out. Foreshadowing her trademark feistiness, she declared she was, indeed, Mighty Casey, but that *she* did not strike out!

Casey studied theater and performance at the University of San Francisco, then moved to New York to study at the American Conservatory Theater, HB Studios, New York University, and the American Comedy Institute. She performed stand-up comedy at Caroline’s, Gotham Comedy Club, Catch A Rising Star, and the New York Comedy Club. Pivoting to broadcast news and sports, she worked as a field producer and engineer for NBC News, where she covered stories for Dateline and Today, political campaigns, wars, NFL Playoff games, Stanley Cup hockey, and the NBA.

Quinlan later moved to Virginia, where she started Quinlan Media Services. She subsequently became VP of Marketing and Operations for Skywire Uplink and launched Mighty Casey Media, LLC. She was awarded the ABWA Richmond Region Business Woman of the Year in 2006 and the Toastmasters International Distinguished Toastmaster Award in 2007. The following year, she was diagnosed with breast cancer. After her treatment, she devoted her business and personal energies to health care advocacy and never looked back.

In 2009, she published a book about her experience, *Cancer for Christmas: Making the Most of a Daunting Gift* [5]. A description of the book on the Amazon website reads [6]:

“Whoopee – cancer!” That’s not your average reaction to a cancer diagnosis, and Casey Quinlan isn’t your average patient. When, after her 15th mammogram, she won the booby prize – breast cancer – her first reaction, after downing a stiff drink, was to cover her own cancer story with the same relentless inquiry she brought to her career in network television news, and that informs her work as a ‘business storyteller’ and branding consultant. Casey’s approach to treatment: be an active participant, not a passive consumer. Her metaphor for managing treatment? “It’s like a car wash. When you go to a car wash, do you want to be inside the car, or strapped to the hood? Ask questions, make sure you understand the answers – you get to stay inside the car. Otherwise, you get lots of soap and wax up your nose!”

## Active Participation in Care

Casey was a thought leader, speaker, and all-around maker of “good trouble.” She was armed with the knowledge—accrued over decades by academics and quality improvement experts—that the *power of patients* could transform and improve health care quality, efficiency, and effectiveness, and lead to better outcomes and patient satisfaction [7-9].

She espoused the principles of participatory medicine, co-design and co-production, where stakeholders do not merely recognize but embrace patients’ and caregivers’ contributions. Riding the wave of the digital era and the democratization of health data, Casey implored people to study their conditions and treatments, examine clinician quality, and engage peers to learn about their experiences. She gained considerable experience in how patient contributions produced greater autonomy and sense of control. She published the following on the Society for Participatory Medicine’s e-Patients Blog [10]:

Patient means different things, to different people, at different times. Whatever anyone’s view of being a patient is, we all have one goal: that others on my care team and around me will respect my definition of my status and seek to understand what it means to me. It can be a role that comes and goes, and returns, different than before, or similar. It can be part of my identity – something felt and lived strongly or coexisting quietly. This too, can develop and change. It can be a view others have toward me, whether I share their view or not. It can mean I’m highly dependent on others (I’m anesthetized for surgery) or highly independent (I’m self-managing) or co-dependent (we’re co-managing).

While a collaborative approach fosters trust and mutual respect, Casey keenly understood that patient-clinician interactions are a tricky dance. She wanted patients to challenge health professionals to be meaningful partners in their care and to gain power in medical decision-making. She expressed the following in her book [5]:

I think that, over the centuries, while medicine has been viewed as a calling, some doctors have misunderstood their place in the doctor-patient relationship. We’re partners in our care. People – again - the ones called “patients” in this party, are not worshipers at the altar of medical professional knowledge, nor are we lesser beings because we don’t have MD behind our names. I suggest that each and every doctor on the planet invest in some communication training, for themselves and their staff. Remember, you’re the partner in your own care.

In December 2014, Consumer Reports posted an article [11], “The surprising way to stay safe in the hospital,” summarizing the results of a survey of 1200 people who had been recently hospitalized. Patients who said they received respectful treatment from clinicians also reported fewer medical errors and better experiences during their hospital stays. Considering this report, Casey blogged [12]:

Engagement may be the buzzword, but accountability is the watchword, for both clinical teams and patients. We all have to participate. Which we can only do if we’re fully informed. There is a good and a bad way of challenging your doctor. The notion that ‘you are the expert when it comes to your body and the doctor is the expert when it comes to medicine’ is a good rule of thumb. There should be a spirit of teamwork that includes shared observations, knowledge and information and asking questions – but not making accusations.

## Getting Your Data and Customizing Evidence

Information is power, and Casey exhibited unparalleled demand for patients and caregivers to have ready access to their electronic health record data. With courage and creativity, she tattooed a QR code on her upper chest which, for a while, opened digital access to her personal record data [13]. She first posted about this in 2015 [14]:

Why did I do this? Because I’ve been waiting for the medical-industrial complex to deliver on their promise of health information exchange (HIE), the promise that they’ve been making for years, but have yet to fork over. I can, and do, securely move money around the globe at the click of a mouse. I do it via bank accounts, purchase agreements, contracts with clients. Most people do. But my healthcare record — which is MINE, as much as it is the property of the medical providers who gave the care it describes — is in fractured bits and pieces all over everywhere.

**Figure 1:**
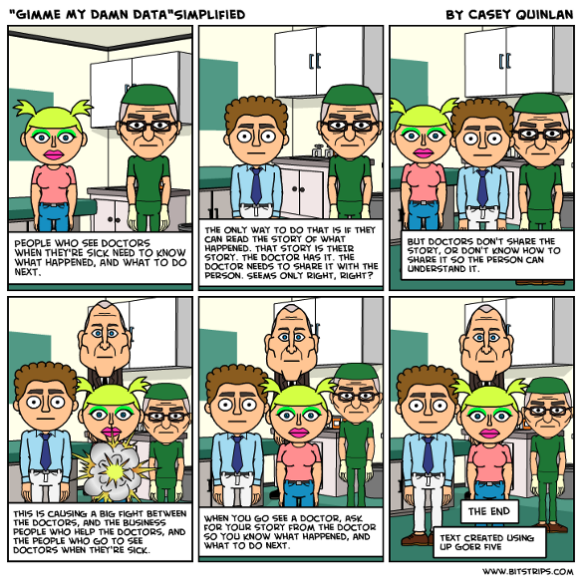
“Gimme My Damn Data - Simplified,” a cartoon by Casey Quinlan (reproduced from Mighty Casey Media [19], which is published under Creative Commons 4.0 Non-Commercial License [20]).

As a patient activist, Casey used all communication channels to implore patients to access their full health records, including cartoons demonstrating this plea (Figure 1). She wrestled with professionals disparaging patients who used the internet, often referred to as “Dr Google” [15]. She argued that well-informed people ask better questions and choose treatments aligned with their values and preferences. Yet, she also knew publicly sourced information could be unreliable and appreciated that searching may produce anxiety or delay seeking professional advice. She was passionate about reputable, evidence-based knowledge generation, and contributed to the efforts of the Cochrane Collaboration, a robust source of high-quality systematic reviews. She actively promoted the Cochrane Consumer Network, a network anyone interested in high-quality evidence can join [16]. She wrote [17]:

Cochrane popped up on my radar screen sometime in the last decade or so, during the time I was scrambling to get on top of managing my parents’ care in the last few years of their lives. It came in handy as I was sifting through my decision tree during cancer treatment ten years ago, and as I’ve become more and more interested in killing off quackery and over-, under-, and mis-treatment in medicine in my work as a citizen science activist and ground-level health policy wonk. We’re all in this together, and Cochrane can help us move the needle toward what I call “Goldilocks medicine” – the right treatment for the right patient, at the right time – at a faster rate.

She was invited to speak at the 2018 Cochrane Collaboration international meeting and coauthored a paper that promoted consumer access to trustworthy evidence customized to patient preferences and contexts [18]. The authors reasoned that “the audience for high quality evidence is much wider than merely health care professionals – and that there is a case to be made for creating tools that translate existing evidence into tools to help patients and clinicians work together to decide next steps.” From this work, Casey continued to argue for greater public access to research locked behind “paywalls” and for patients and families to provide more genuine and robust contributions to decision support tools.

## Patients Are Experts: Ignore Them at Your Peril!

Casey’s trademark humor and incisive analysis of the health care system made her a highly sought-after speaker and consultant. She was honored with the Right Care Alliance Leadership Award (2017) and the WEGO Health Best One Liner Award. She served on multiple boards and steering committees, notably the Society for Participatory Medicine, Health Datapalooza, and the Light Collective. She worked tirelessly to raise awareness about the needs and rights of all patients and particularly encouraged metastatic breast cancer research. She served as an expert patient and exemplar of co-design at health technology events and passionately helped drive patient and consumer attendance at health industry and scientific conferences [10]:

If you’re planning a healthcare industry event that is focused on patient engagement, patient-centered design, patient-centered care, patient-centered technology, or touches on patient care in any part of the healthcare setting or system, you have to include patients on your program or be judged Patients Excluded. Nothing about us without us.

Casey advocated for compensating patients and caregivers for their time spent informing health care system improvement, whether on advisory boards, speaking engagements, or providing feedback, remarking that “warm handshakes and cold bagels” were insulting. Instead, Casey advocated for fair payment models for patients and caregivers—who she called *ground level experts*.

Casey also felt that all revenue generated from monetizing patients’ personal data should be shared with the people who are, after all, she reasoned, the source of such data. In a blog post, she elaborated [21]:

Let's take to the streets, the halls of Congress and state capitals, policy meetings, and star-chambers from sea to shining sea, to demand compensation for the ginormous wads of cash that are minted—literally—from our bodies, bones, blood.

## A Unique Role Model

Casey’s outspoken nature could be intimidating until one got to know her. She took time to listen. She supported and encouraged many others to find their voices and tell their stories. Her courage and determination left an indelible legacy of advocacy for a more equitable and effective health care system. She approached her cancer as intensely as she lived her life. Friends, colleagues, and loved ones found solace in the fact that her principles and values remained evident even at the end of her life. During a Health Hats podcast interview [22], she retorted:

So, f**k cancer, I’m not done, and I’m not quitting until I'm dead. And then I want you all to carry me off the battlefield on my shield and then keep fighting. Because that's the only way we're going to hack this universe into a more human-friendly place.

Casey was a role model who gave us the courage to speak up, for ourselves and for all those affected by bureaucracy, inequity, and arrogance. Today, as artificial intelligence (AI) garners much attention, we can imagine Casey front and center, promoting the promise of AI and large language models to sharpen patients’ engagement with decision-making, while cautioning us all about its risks. Her irreverence, prominently displayed in the name of her podcast, Healthcare is Hilarious!, admonished us to embrace joy as we figure it all out [5]:

My philosophy in a nutshell: Life is 100% fatal. Let’s have fun while we’re here.

## Abbreviations

AI: artificial intelligence
